# *Tribbles* Genes in Gastric Cancer: A Tumor-Suppressive Role for *TRIB2*

**DOI:** 10.3390/genes15010026

**Published:** 2023-12-23

**Authors:** Alessia Foscarini, Rossella Tricarico, Federica Gentile, Swapna Satam, Hermine Mohr, Endre Kiss-Toth, Guglielmina Nadia Ranzani, Natalia Simona Pellegata

**Affiliations:** 1 Department of Biology and Biotechnology “L. Spallanzani”, University of Pavia, 27100 Pavia, Italy; alessia.foscarini01@universitadipavia.it (A.F.); rossella.tricarico@unipv.it (R.T.); federica.gentile01@universitadipavia.it (F.G.); 2 Institute for Diabetes and Cancer, Helmholtz Munich, 85764 Neuherberg, Germany; ssatam@gmail.com (S.S.); hermine.mohr@helmholtz-munich.de (H.M.); 3 Division of Clinical Medicine, School of Medicine and Population Health, University of Sheffield, Sheffield S10 2TN, UK; e.kiss-toth@sheffield.ac.uk

**Keywords:** gastric cancer, Tribbles, TRIB2, CIN phenotype, tumor suppressor

## Abstract

Tribbles pseudokinases (TRIB1-3) are important signaling modulators involved in several cancers. However, their function in gastric cancer (GC) remains undefined. GC is still a deadly disease since the lack of sensitive and specific biomarkers for early diagnosis and therapy response prediction negatively affects patients’ outcome. The identification of novel molecular players may lead to more effective diagnostic and therapeutic avenues. Therefore, we investigated the role of *TRIB* genes in gastric tumorigenesis. Data mining of the TCGA dataset revealed that chromosomal instability (CIN) tumors have lower *TRIB2* and higher *TRIB3* expression versus microsatellite instability (MSI)-high tumors, while *TRIB1* levels are similar in both tumor types. Moreover, in CIN tumors, low *TRIB2* expression is significantly associated with aggressive stage IV disease. As no studies on *TRIB2* in GC are available, we focused on this gene for further in vitro analyses. We checked the effect of *TRIB2* overexpression (OE) on MKN45 and NCI-N87 CIN GC cell lines. In MKN45 cells, *TRIB2* OE reduced proliferation and colony formation ability and induced G2/M arrest, while it decreased the proliferation and cell motility of NCI-N87 cells. These effects were not mediated by the MAPK pathway. Our results suggest a tumor-suppressive function of *TRIB2* in GC with a CIN phenotype.

## 1. Introduction

Gastric cancer (GC) is the fifth most common cancer and the fourth leading cause of cancer mortality in both sexes worldwide [1]. Given that in early stages, GC is frequently asymptomatic, patients are often diagnosed at an advanced stage, negatively affecting disease outcome. To increase patients´ burden, no specific and sensitive biomarkers are available to assist early GC diagnosis [2]. Therapy options depend on tumor stage. For instance, in localized disease, the only curative option is prophylactic gastrectomy. In advanced stages, systemic treatment options are the standard of care [3]. Considering its beneficial effects on survival and favorable safety profile, the preferred first-line therapy is a combination of fluoropyrimidine (e.g., 5-Fluorouracil—5FU) and platinum-based agents (cisplatin or oxaliplatin). However, resistance to chemotherapy is common, and this ultimately leads to treatment failure [4]. In this scenario, the identification of novel molecular biomarkers is needed to improve a timely diagnosis, as well as for the discovery of novel therapeutic targets that may lead to effective therapies.

The great majority (90–95%) of GCs are adenocarcinomas that include two main histological subtypes, intestinal (well-differentiated) and diffuse (undifferentiated) types, according to Lauren’s classification [5]. Lauren’s criteria, although introduced some 60 years ago, remain currently accepted since they represent a simple and robust classification system. Moreover, accumulating evidence over time has shown that intestinal and diffuse GCs exhibit differing etiology and pathogenesis, carcinogenesis, biological behaviors, and prognosis. This classification, however, fails to reflect the great molecular and genetic heterogeneity of GCs which is of clinical relevance to identify novel targets for prevention and treatment. 

In recent years, several molecular classifications have been proposed to relate GC histopathological and molecular characteristics to clinical features. The Cancer Genome Atlas project analyzed 295 primary gastric adenocarcinomas with the purpose of defining clinically relevant molecular subgroups [6]. This study identified four subtypes: tumors with chromosomal instability (CIN), accounting for 49.8% of all cases and characterized by extended aneuploidy and chromosomal instability; tumors with microsatellite instability (MSI), which represent 21.7% of cases and are characterized by a MSI-high (H) status; genomically stable tumors (GS), representing 19.7% of cases; Epstein–Barr virus (EBV+) positive tumors, which represent 8.8% of all GCs and are characterized by a CpG island methylator phenotype (CIMP). More recently, EBV and GS subtypes have been associated with the best and with the worst prognosis, respectively, while patients with MSI and CIN tumors have been reported to show poorer overall survival than EBV but better than GS patients [7].

The *Tribbles* (*TRIB*) gene family encodes a group of highly conserved serine/threonine pseudokinases (TRIB1-3). They contain the substrate binding-domain of protein kinases but do not possess ATP binding sites nor catalytic motifs essential for kinase activity. TRIB proteins regulate the activity and the degradation of transcription factors and other signaling proteins, and act as scaffolds able to modulate the MAPK and AKT pathways [8]. Consequently, these proteins play a role in cell cycle, differentiation, cell proliferation, cellular metabolism, and stress, in both physiological and pathological conditions [9,10]. In cancer, *TRIB*s can behave either as oncogenes or tumor suppressors depending on the gene and on the cellular context [11]. *TRIB1*, *TRIB2,* and *TRIB3* have been involved in different cancers including hematological malignancies (mostly *TRIB2*) and a variety of solid tumors [12].

A few studies have investigated the role of *TRIBs* in GC, mostly focusing on the *TRIB3* gene. Its expression was associated with either worse or more favorable prognosis, depending on the study [13,14], and was suggested to have predictive value in the prognostic stratification of GC patients [15]. So far, there is one only study on *TRIB2* in GC, reporting that its level is downregulated in MGC-803 GC cells following treatment with the anti-cancer agent dioscin [16]. No data are available for *TRIB1* in GC.

In this study, we first systematically explored the pattern of genetic and genomic alterations of *TRIB* genes in GC tissues by mining the Stomach Adenocarcinoma (STAD) TCGA dataset. We then extended our in silico analysis to the mRNA expression profile of the three genes, which was correlated with clinical parameters. Based on the results of this in silico approach, we then focused on investigating the role of *TRIB2* in GC using in vitro experimental models of CIN GC and functional assays. We here report that *TRIB2* behaves as a tumor suppressor gene in CIN GC.

## 2. Materials and Methods

### 2.1. Databases

For the in silico analysis, we performed data mining of The Cancer Genome Atlas (TCGA) dataset—Stomach Adenocarcinoma (STAD) dataset publicly available on the cBio Cancer Genomics Portal (cBioPortal: https://www.cbioportal.org/; v3.6.9; accessed on 2 February 2021) and on the UALCAN database (https://ualcan.path.uab.edu/analysis.html; accessed on 22 February 2021). We also investigated the transcriptomic profiles of a panel of gastric cancer cell lines available on the Cancer Cell Line Encyclopedia (CCLE) database (hosted on the Cancer Dependency Portal (DepMap; https://depmap.org/portal/; accessed on 8 February 2021)

### 2.2. Bioinformatics Analysis

mRNA expression data, expressed as z-scores (log RNA seq V2 RSEM) and downloaded from cBioPortal, were normalized based on all TCGA profiled samples and plotted to obtain a distribution curve for each *TRIB* gene. The distribution values were divided into quartiles, using the Excel’s INC.QUARTILE function: the first quartile (high-25) included the highest expression values (n = 103), whereas the fourth quartile (low-25) included the samples with the lowest expression (n = 103). Based on this division, cut-offs corresponding to the threshold value of the quartiles were defined: for both *TRIB1* and *TRIB2*, the thresholds were +0.6 and −0.6 for the first and fourth quartiles, respectively; for *TRIB3,* the thresholds were +0.7 and −0.7 (see Figure S1). The thresholds correspond to the values of z-scores indicated on the cBioPortal. Based on preliminary tests, where the expression level of each gene was divided into various percentiles, we chose to use the threshold value between the first and second quartiles as the cut-off to compare the high-25 with all other samples (others = intermediate/low expression). By comparing “high-25” (n = 103) versus “others” (n = 309), we could maintain the entire dataset and thus derive information from 412 samples per gene (see Figure S2).

This approach was individually applied to each molecular subtype belonging to the STAD dataset. Once the cut-offs were defined, bioinformatics gene expression analyses were re-run on cBioPortal for STAD, according to the described procedure.

### 2.3. Gastric Cancer Cell Lines

We employed two human GC cell lines: MKN45, derived from a liver metastasis of poorly differentiated gastric adenocarcinoma (diffuse histotype) and purchased from the Cell Bank RIKEN BioResource Center (Tsukuba, Japan); NCI-N87 cells, derived from a liver metastasis of a well-differentiated carcinoma of the stomach (intestinal histotype) and kindly provided by Dr. D. Calistri (IRST-IRCCS, Meldola, FC, Italy). The two selected cell lines were microsatellite stable (MSS) with a CIN phenotype. Cells were cultured in RPMI1640 media (Gibco-Thermo Fisher Scientific, Waltham, MA, USA) supplemented with 10% FBS. HEK293T cells were cultured in DMEM (Gibco-Thermo Fisher Scientific) supplemented with 10% FBS. All media were supplemented with 100 units/mL penicillin–streptomycin (Thermo Fisher Scientific) and 1 mg/mL Primocin (Invivogen, San Diego, CA, USA), and cells were grown at 37 °C in a humidified 5% CO_2_ incubator. Cell lines were authenticated by the Leibniz Institute DSMZ when obtained and were regularly tested for mycoplasma detection by PCR (PCR Mycoplasma Test Kit I/C, Promocell, Heidelberg, Germany).

### 2.4. Lentiviral-Mediated Gene Modulation

MKN45 and NCI-N87 cells were transduced with lentiviral vectors to obtain stable overexpression (OE) of TRIB2 or the control green fluorescent protein (GFP). The vectors for GFP (pLenti-C-mGFP-P2A-Puro) and TRIB2 (pLenti-C-mGFP-P2A-Puro-TRIB2) overexpression were purchased from OriGene Technologies GmbH (Herford, Germany). These vectors were used to produce GFP OE and TRIB2 OE lentiviruses in the HEK293T cells. HEK293T cells were seeded in 10 cm culture dishes at a density of 1 × 10^6^ cells/plate, and the calcium phosphate transfection was performed after 24 h, when cells reached about 80% confluence. Transfection complex solution comprising 6 µg psPAX2 and pmD2.G packaging vectors, 5 µg lentiviral plasmid, CaP (calcium phosphate), HBS (HEPES-buffered saline) buffer, and H_2_O was added to the culture dish with HEK293T cells, according to the manufacturer’s protocol. After 24 h, the culture medium was replaced with DMEM containing 10% FBS. The supernatant of HEK293T cells was collected 48 h after the transfection and the lentiviruses were filtered by a 0.45 μm filter. Lenti-X GoStix Plus (Takara Bio, Kusatsu, Japan) was used to determine the titer of the lentiviruses. The obtained lentiviral vectors were used to deliver TRIB2 as the GFP fusion protein or the GFP control protein alone to MKN45 and NCI-N87 cells.

MKN45 and NCI-N87 cells were seeded at 5 × 10^5^ cells/well in 6-well plates. After 24 h, 2 mL lentivirus and 1 µg/mL Polybrene (Sigma Aldrich, St. Louis, MO, USA) were added into each well. After 6 h, the culture medium was replaced with RPMI-1640 medium containing 10% FBS. After 72 h, culture medium containing 1 µg/mL Puromycin was used to select the cells for about 2 weeks to obtain stable transfected cell lines.

The cell lines were transduced with the TRIB2 OE vector or the GFP OE vector used as the control. The OE of TRIB2 in the stable cell lines was confirmed by Western blot analysis.

### 2.5. Western Blotting

The cells were harvested and lysed in RIPA lysis buffer (Sigma Aldrich) supplemented with a protease and phosphatase inhibitor cocktail (Sigma Aldrich). Protein extracts were run on 10% SDS-polyacrylamide gel electrophoresis and transferred to nitrocellulose membranes by voltage gradient transfer. The blots were blocked for 1 h at room temperature in 5% non-fat dry milk. The membranes were then incubated overnight at 4 °C with the primary antibodies for TRIB2 (ATLAS ANTIBODIES, Bromma, Sweden), for AKT, Phospho-Akt (Ser473), ERK, and Phospho-ERK (137F5) (Cell Signaling Technology, Danvers, MA, USA) diluted at 1:1000, and then incubated with an anti-rabbit secondary antibody (Sigma-Aldrich). The visualization of TRIB2 protein bands was obtained by using the horseradish peroxidase (HRP) development system.

### 2.6. Western Blot Quantification Using ImageJ

The obtained final images of the Western blots were analyzed by the ImageJ software program (version 2.14.0/1.54f) to quantify the intensities of the protein bands by the IntDen value (mean gray values × pixel number). All of the quantifications were normalized to the expression of the control protein α-tubulin.

### 2.7. Cell Viability Assay

Cells were seeded into a 96-well plate with 5000 cells/well for MKN45 and 10,000 cells/well for NCI-N87 cells. The cell viability was measured using the Cell Proliferation Reagent WST-1 (Roche, Basel, Switzerland): at different time points, 10 µL WST-1 (water-soluble tetrazolium salt) was added into each well and the absorbance was measured at 440–450 nm every 30 min, every day for 4 days. Each time point had six replicates.

### 2.8. Colony Formation Assay

The ability of the cells to form a colony was tested by seeding MKN45 and NCI-N87 cells in 6-well plates with 1000 cells/well incubated for 10 days. At this time point, the colonies in each well were stained by crystal violet and then counted with countPHICS program.

### 2.9. Cell Cycle Analysis

For each cell line, 5 × 10^5^ cells were seeded in a medium flask (75 cm^2^), and after 24 h of starvation, the FBS-free medium was replaced with normal medium for 24 h, 48 h, or 72 h. At each time point, the cells were harvested and resuspended in 75% cold ethanol, treated with 10 μg/mL RNAse at 37 °C for 15 min, and stained with 50 μg/mL Propidium iodide (PI) solution for 1 h. Cell cycle analysis was carried out by comparing the parental cells with the stably infected cells expressing GFP (control) or TRIB2. Then, the samples were analyzed by MACSQuant Analyzer Flow Cytometry (Miltenyi Biotec—Bergisch Gladbach, Germany) and the obtained data were analyzed by FlowJo program.

### 2.10. Migration Assay

Cell migration was determined via the transwell method using ThinCerts^®^ inserts with a size of pore of 8 μm (Greiner Bio-One, Kremsmünster, Austria). A total of 1.5 × 10^4^ cells were seeded without serum in the upper part of the ThinCerts^®^ inserts. After 24 h, the lower insert was placed in complete medium with 10% FBS. Cells were then incubated for 24 h and 48 h. At the different time points (24 h and 48 h), cells were stained by hematoxylin, and cell count was performed using the Fiji plugin Cell Counter.

### 2.11. Drug Treatment

5-Fluorouracil (5-FU) was purchased from Sigma-Aldrich. Cells were seeded into a 96-well plate with 5000 cells/well for MKN45 and 10,000 cells/well for NCI-N87 cells. After 24 h of incubation, cells were treated with various concentrations of 5-FU (50, 100, 200, and 400 μM) for 72 h. The cytotoxicity was evaluated by cell viability assay as described above. Determination of the drug concentration to inhibit cell proliferation by 50% (IC50) was calculated for each cell lines based on the parental cells.

### 2.12. Statistical Analysis

*p* values were calculated with the Mann–Whitney statistical test, Welch’s test, and multiple T tests with GraphPadPrism v8.0 software. Statistical significance was defined as * *p* < 0.05; ** *p* < 0.01; *** *p* < 0.001; **** *p* ≤ 0.0001. Each graph is representative of multiple independent experiments. Data are shown as mean  ±  standard deviation (SD).

## 3. Results

### 3.1. TRIBs Genetic and Genomic Somatic Alterations Are Infrequent Events in GC

**Figure 1:**
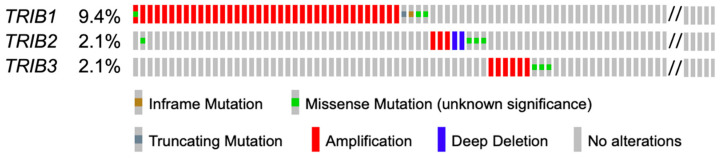
*TRIBs* alterations in the TCGA PanCancer Atlas dataset. Frequency and types of genetic and genomic alterations of the 3 genes in stomach adenocarcinoma (STAD). Each small rectangle represents a sample. The figure is a partial graph highlighting samples with alterations.

We focused on the frequency and type of genetic and genomic alterations of TRIB genes in GC by analyzing the Stomach Adenocarcinoma (STAD) samples included in the TCGA PanCan Atlas dataset. To note, *TRIB1* is localized at the cytogenetic band 8q24.13; *TRIB2*, at 2p24.3; *TRIB3*, at 20p13. In STAD, the total frequency of alterations of all *TRIB* genes was 13.6% (59/434). Specifically, alterations in *TRIB1*, *TRIB2,* and *TRIB3* were present in 9.4% (41/434), 2.1% (9/434), and 2.1% (9/434) of GC cases, respectively. The most frequent alteration was gene amplification (mostly of *TRIB1*), while deletions and point mutations were quite rare (Figure 1).

### 3.2. In CIN GC, Low TRIB2 mRNA Expression Correlates with Advanced Tumor Stage

**Figure 2:**
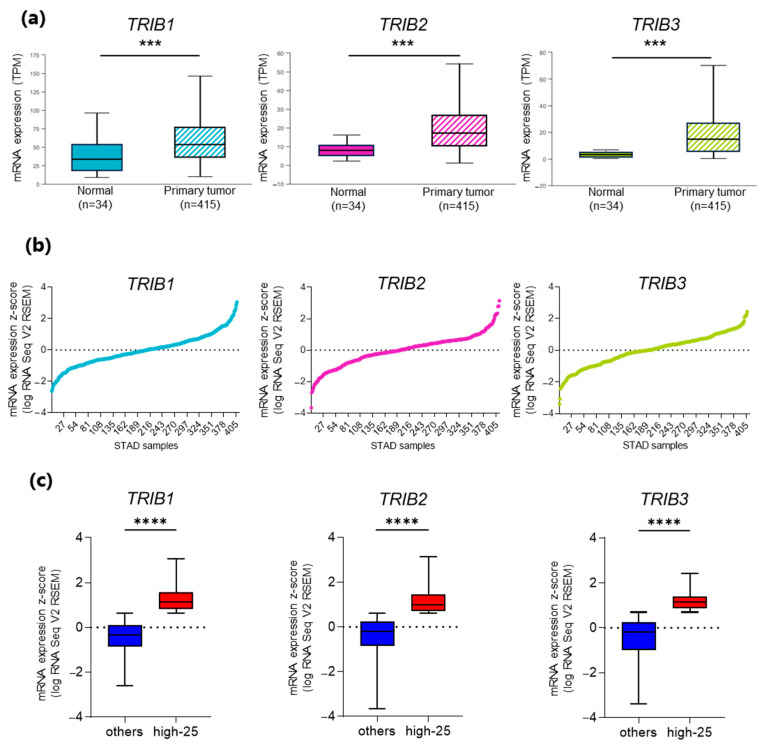
*TRIB1*, *TRIB2,* and *TRIB3* mRNA distribution profile. (**a**) Expression levels of *TRIB1*, *TRIB2,* and *TRIB3* genes in normal and cancer (STAD) tissue samples from UALCAN database. The *Y*-axis shows mRNA levels expressed in transcripts per million (TPM). (**b**) *TRIB1*, *TRIB2,* and *TRIB3* mRNA level distribution profile in *TCGA PanCan STAD* dataset from cBioPortal database. The *Y*-axis shows mRNA levels expressed in z-scores (log RNASeq V2 RSEM). (**c**) Box plots showing a significant difference in mRNA expression levels between quartiles (“high-25” vs. “others”) for *TRIB1*, *TRIB2,* and *TRIB3*. The *Y*-axis shows mRNA levels expressed in z-scores (logRNASeq V2 RSEM). Data are shown as mean ± standard deviation (SD). *** *p* < 0.001; **** *p* ≤ 0.0001.

To investigate *TRIBs* gene expression in normal and tumor gastric tissues, we performed data mining of the TCGA STAD dataset available on UALCAN and we compared the mRNA expression level of the *TRIB* genes in normal (n = 34) and tumor (n = 415) samples. *TRIB1*, *TRIB2,* and *TRIB3* showed a significantly higher expression in tumors compared to normal tissue samples (Figure 2a). Moreover, we analyzed the distribution profile of *TRIB* mRNAs in the 415 TCGA STAD tumors in cBioPortal. We found that mRNA expression levels of *TRIBs* were highly variable among samples (Figure 2b).

To correlate the level of the *TRIB* genes with the molecular and clinical characteristics of GC patients, we stratified the samples into quartiles based on the expression levels of each gene. The strategy of sample stratification is reported in Materials and Methods and the results are shown in Figures S1 and S2. When comparing, for each gene, the set of samples showing the highest expression levels (i.e., the 103 samples of the first quartile: “high-25”) and the set of samples showing intermediate/low expression levels (i.e., the 309 samples of the three other quartiles: “others”), the difference between the two groups proved to be highly significant (Figure 2c). Therefore, subsequent correlation studies were conducted by comparing “high-25” and “others” subsets for each gene.

**Figure 3:**
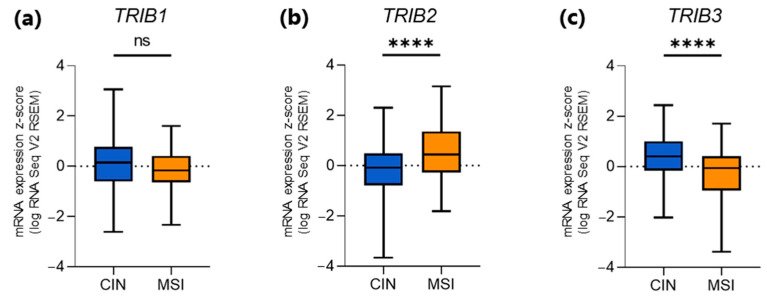
Box plots showing *TRIB1* (**a**), *TRIB2* (**b**), and *TRIB3* (**c**) expression in STAD tumors with CIN or MSI phenotype. The *Y*-axis shows mRNA level expressed in z-scores (log RNASeq V2 RSEM). Data are shown as mean ± standard deviation (SD); **** *p* ≤ 0.0001; ns, not significant.

We first investigated whether the level of expression of the *TRIB* genes (high vs. intermediate/low) correlated with the molecular subdivision of tumors in chromosomally unstable (CIN; n = 223), microsatellite unstable (MSI; n = 73), genomically stable (GS; n = 50), Epstein–Barr virus positive (EBV; n = 30), and *POLE* mutated (POLE; n = 7) subtypes. Overall, each molecular subtype included cases with high and intermediate/low expression level, although in different proportions (see Table 1). When only considering the two most common GC molecular subtypes, i.e., CIN and MSI tumors, a significantly lower expression of *TRIB2* was found in CIN vs. MSI tumors (*p* < 0.0001) (Figure 3b), whereas *TRIB3* was more highly expressed in CIN tumors compared to MSI cases (*p* < 0.0001) (Figure 3c). No association between *TRIB1* mRNA expression and a CIN/MSI phenotype was found (Figure 3a).

**Figure 4:**
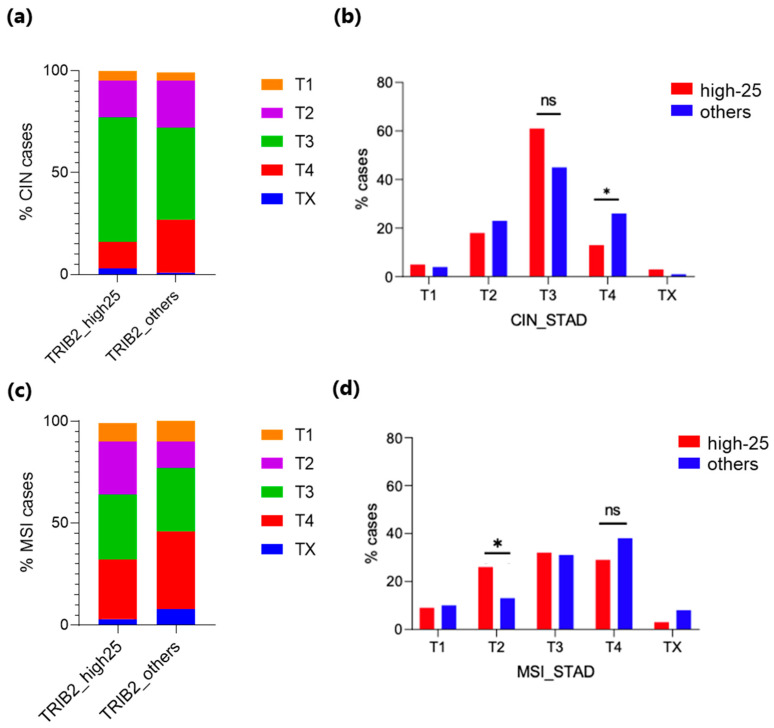
Distribution of CIN (**a**,**b**) and MSI (**c**,**d**) tumors in the TCGA-STAD database belonging to different tumor stages and having high (“high-25”) or low/intermediate (“others”) expression of *TRIB2*. T1 to T4: less advanced to more advanced stage; TX: undetermined stage. * *p* < 0.05; ns, not significant.

Studies assessing the prognostic value of *TRIB3* overexpression, albeit controversial, have been reported [13,14,15], as well as work on its anti-apoptotic role in doxorubicin-treated GC cell lines [17]. In contrast, the *TRIB2* gene had not already been investigated for its role in GC. Therefore, we focused on *TRIB2* for further analyses. We correlated *TRIB2* gene expression (high vs. low/intermediate) with patients’ sex, tumor histologic grade, and tumor stage. We correlated *TRIB2* gene expression (high vs. low/intermediate) with patients’ sex, tumor histologic grade, and tumor stage. Males and females were similarly represented in CIN tumors showing high *TRIB2* gene expression (females: 40%, 15/38; males: 60%, 23/38) and low/intermediate expression (females: 30%, 56/185; males: 70%, 129/185). In MSI tumors, females were more prevalent in the low/intermediate *TRIB2* mRNA expression subset (64.1%, 25/39) compared to males (35.9%, 14/39), while males were more prevalent in the highest *TRIB2* mRNA expression subset (61.8%, 21/34) compared to females (38.2%, 13/34) (Figure S3a). In both CIN and MSI tumors, no significant correlation was observed between *TRIB2* mRNA expression levels and histological grade (Figure S3b). Interestingly, in the CIN subset, advanced T4 stage tumors more often showed low/intermediate levels of *TRIB2* expression (T4; *p* = 0.0374), while in the MSI subset, the majority of early T2 stage tumors were characterized by a high *TRIB2* expression (*p* = 0.0374) (Figure 4).

### 3.3. TRIB2 Overexpression Suppresses the Growth of GC Cell Lines with a CIN Phenotype

As shown above, *TRIB2* is less expressed in CIN vs. MSI GCs, and its expression level is lower in CIN tumors of advanced stage. This suggests that *TRIB2* might play a tumor-suppressive role in GCs with a CIN phenotype, its downregulation being advantageous for tumor progression. To explore the potential role of *TRIB2* in CIN gastric tumorigenesis, we performed in vitro functional assays.

**Figure 5:**
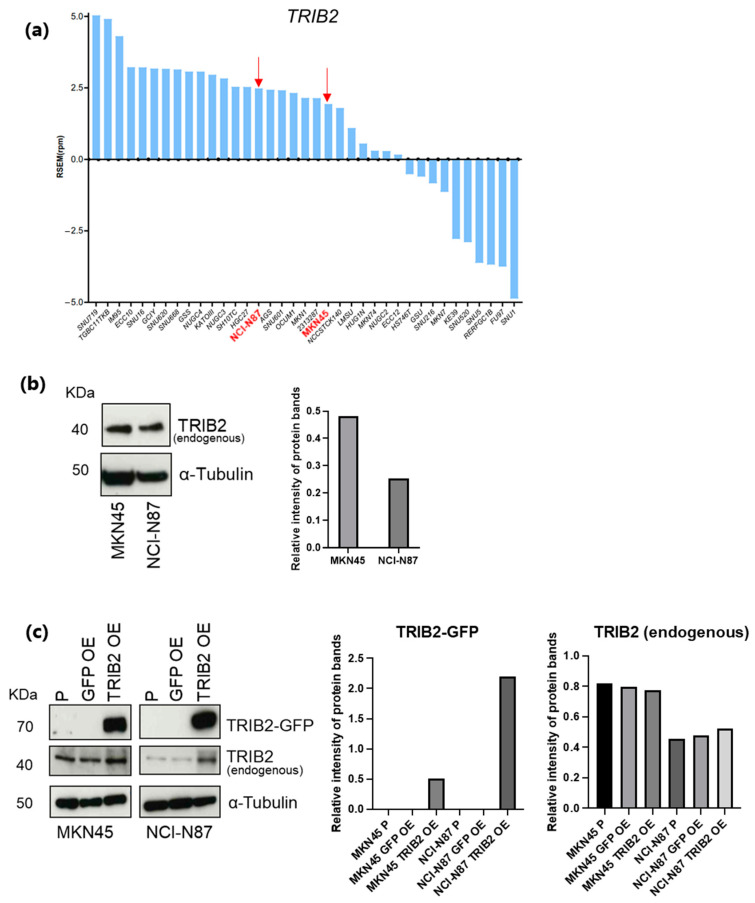
Modulation of TRIB2 expression in CIN GC cell lines. (**a**) Bar graph representing the expression level of *TRIB2* mRNA in GC cell lines included in the CCLE database. MKN45 and NCI-N87 cells are highlighted in red and bold as they were selected for subsequent experiments. (**b**) Western blot (WB) showing the endogenous expression of TRIB2 protein in MKN45 and NCI-N87 cells. α-tubulin was used as internal control. The right panel reports the quantification of the bands normalized to α-tubulin. (**c**) WB showing endogenous and ectopic (fused with GFP) TRIB2 protein expression in untransfected parental (P), GFP overexpressing (GFP OE), and TRIB2 overexpressing (TRIB2 OE) MKN45 and NCI-N87 cells. α-tubulin was used as internal control. The panels on the right illustrate the quantification of the bands normalized to α-tubulin.

We observed that GC cell lines included in the CCLE database are characterized by quite different *TRIB2* expression levels (Figure 5a). To perform in vitro assays, we selected MKN45 and NCI-N87 cells that have a CIN phenotype and “intermediate” *TRIB2* gene expression. We confirmed this in silico observation through Western blot (WB), showing that these cells express endogenous TRIB2 protein at detectable levels (Figure 5b).

MKN45 and NCI-N87 cells were infected with lentiviral vectors expressing a TRIB2-GFP fusion protein, or only GFP as a negative control. Stable GC cell lines ectopically overexpressing TRIB2 were selected. In cell lines, the TRIB2 overexpression (OE) was confirmed by WB analysis (Figure 5c).

**Figure 6:**
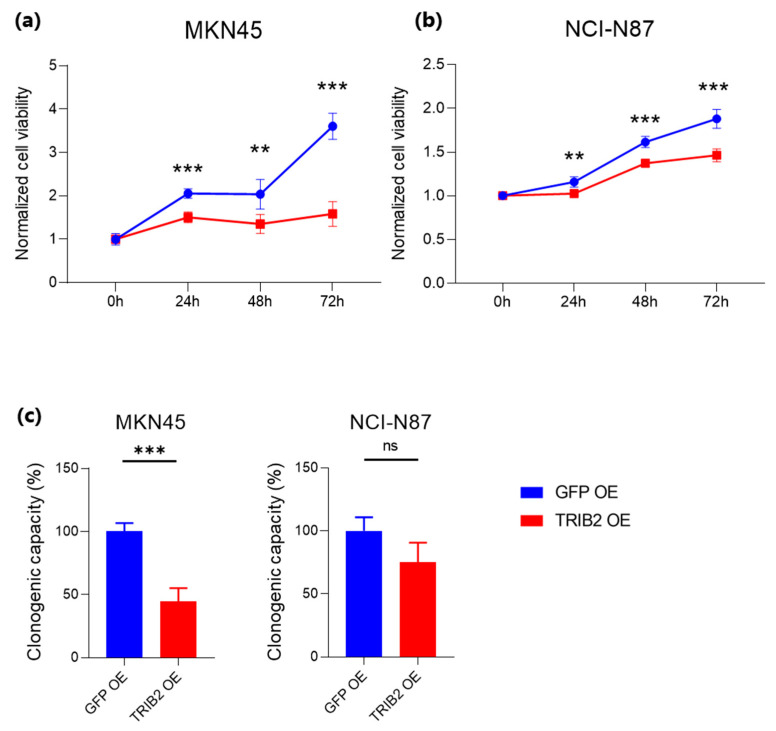
Effect of TRIB2 OE on CIN GC cell proliferation. (**a**,**b**) Proliferation of MKN45 (**a**) and NCI-N87 (**b**) cells overexpressing GFP (control) and TRIB2. Samples were measured in triplicate and shown is the average of 3 independent experiments. (**c**) Cell colony formation assay of MKN45 TRIB2 OE and NCI-N87 TRIB2 OE cells compared to control GFP OE cells. (**a**–**c**) Data are shown as mean ± standard deviation (SD). **, *p* < 0.05; ***, *p* < 0.01; ns, not significant.

We then assessed the effect of TRIB2 OE on relevant oncogenic features, starting with proliferation and colony formation. Our data showed that TRIB2 OE can significantly inhibit the proliferation of MKN45 and NCI-N87 cells vs. the control GFP-infected cells (Figure 6a–c). We next determined the effect of TRIB2 OE on cell growth by conducting colony formation assays. We found that TRIB2 OE significantly reduced the ability of MKN45 cells to form colonies when compared to the control GFP-infected cells (Figure 6c). For NCI-N87 cells, there was a similar trend, which was, however, not significant (Figure 6c).

**Figure 7:**
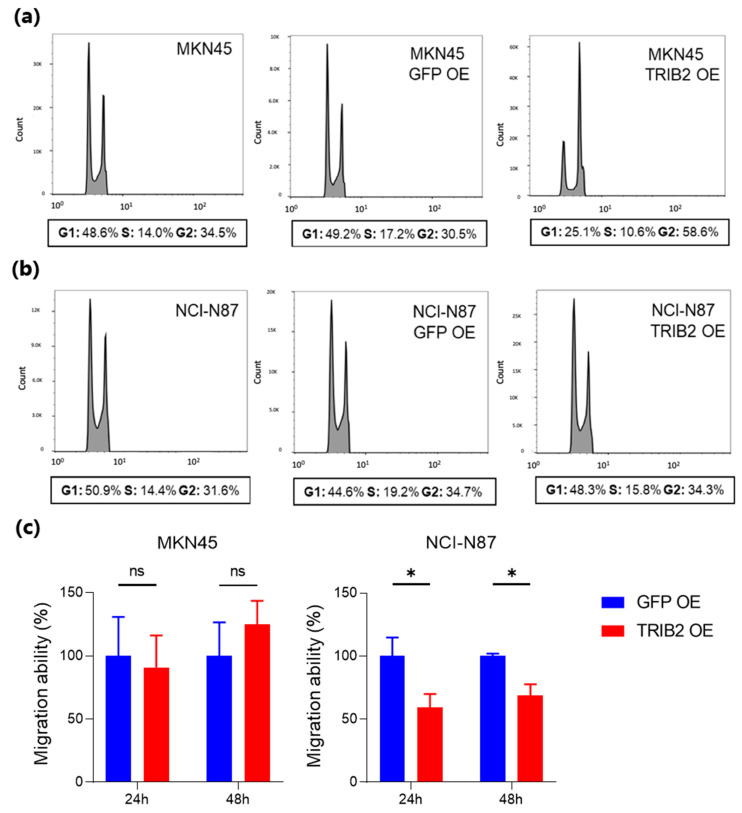
(**a**,**b**) Cell cycle analysis of MKN45 (**a**) and NCI-N87 (**b**) cells either untransfected (parental), overexpressing GFP (GFP OE; controls), or overexpressing TRIB2 (TRIB2 OE). The percentage of cells in the various phases of the cell cycle is reported below the graphs (representative of 3 independent experiments showing the same results). (**c**) TRIB2 OE effect on cell migration. Transwell migration assays on MKN45 and NCI-N87 overexpressing TRIB2 (TRIB2 OE) or overexpressing GFP (GFP OE), the latter used as control cells. Data are shown as mean ± standard deviation (SD). * *p* < 0.05; ns, not significant.

To better characterize the inhibition of cell proliferation upon TRIB2 OE, cell cycle analysis was carried out by comparing parental cells with cells having TRIB2 OE or GFP expression (control cells). The results showed for MKN45 parental cells (not transduced) the following percentages of cells in the various cell cycle stages: G1: 48.6%, S: 14%, G2: 34.5%; MKN45 GFP OE: G1: 49.2%, S: 17.2%, G2: 30.6%; MKN45 TRIB2 OE: G1: 25.1%, S: 10.6%, G2: 58.6%. NCI-N87 parental cells (not transduced) showed the following percentages: G1: 50.9%, S: 14.4%, G2: 31.6%; NCI-N87 GFP OE: G1: 44.6%, S: 19.2%, G2: 34.7%; NCI-N87 TRIB2 OE: G1: 48.3%, S: 15.8%, G2: 34.3%. While TRIB2 OE had no effect on the cell cycle distribution of NCI-N87, it caused G2 arrest in MKN45 cells (Figure 7a,b).

Moreover, we evaluated the migration ability of the TRIB2 OE cell lines. TRIB2-OE did not affect MKN45 cell migration, while NCI-N87 TRIB2 OE cells showed reduced migration capacity compared to GFP control cells (Figure 7c).

In several human cancers, the effect of Tribbles, including TRIB2, were shown to be mediated by their ability to modulate the PI3K and/or the MAPK pathway (see Section 1). To investigate whether an inhibition of these signaling cascades might explain the anti-proliferative effects of TRIB2 in GC cells, we assessed the levels of phosphorylated (=active) AKT and ERK proteins in GFP control and TRIB2 OE cells through WB. As shown in Figure S4, the relative expression levels of the active form of these proteins were similar among parental, GFP control, and TRIB2 OE cells. This indicates that the PI3K and MAPK pathways are not likely to mediate the tumor-suppressive effects of TRIB2 in GC, which is therefore associated to additional downstream effectors.

In various cancer cell lines and primary tumors, e.g., osteosarcoma and melanoma, TRIB2 overexpression has been shown to mediate resistance against 5-FU through the disruption of AKT/FOXO and AKT/MDM2/p53 pathways [12]. Since 5-FU is a commonly used chemotherapeutic agent for GC, we treated the GFP control and TRIB2 OE cells with different concentrations of 5-FU to verify whether TRIB2 regulates the response to this agent. No difference in cell survival was detected between TRIB2 OE and the control GFP OE cells treated for 72 h with 5-FU (Figure S5), suggesting that TRIB2 does not affect the response of MKN45 and NCI-N87 GC cells to the drug.

## 4. Discussion

TRIB proteins have been implicated in different cancer types, where they play either oncogenic or tumor-suppressive roles depending on the family member and the cellular context [12]. Data mining of genetic and genomic alterations of *TRIBs* across tumors of the TCGA dataset revealed that these genes are altered in a subset of cases, with *TRIB1* being the most frequently mutated family member, mainly via amplification. 

Specifically focusing on gastric cancer cases (STAD) within the TCGA, we found a very low frequency of *TRIBs* genetic and genomic alterations (*TRIB1*: 9.4%, *TRIB2*: 2.1%, and *TRIB3*: 2.1%). The most common alteration was *TRIB1* gene amplification, occurring in 8.5% of cases. Previous articles have identified recurrent amplification of various chromosomal regions in primary GC samples. Interestingly, among these regions is the chromosomal band 8q24.13 [18,19], where *TRIB1* is located. This region maps also the transcription factor c*MYC*, a well-known proto-oncogene, which was found to be part of the 8q24.13 amplicon in GC [18]. No studies have assessed whether *TRIB1* is co-amplified together with c*MYC* in GC; however, both genes were found to belong to the same amplicon in prostate cancer [20]. Thus, a similar situation may also apply to GC. Further studies are needed to assess the potential contribution of *TRIB1* amplification to the pathogenesis of a subset of GC. Amplification of *TRIB2* and *TRIB3* occurred in rare cases, and the chromosomal regions where these genes are located were not reported to be amplified in GC.

The expression of the *TRIB* genes in the TCGA GC dataset was highly variable. Upon stratification of GC patients based on *TRIB* mRNA levels, we correlated gene expression with GC molecular subtypes. With regard to the two most common GC subtypes (CIN and MSI), *TRIB1* expression proved to be similar in both groups, while *TRIB2* showed a significantly lower expression and *TRIB3* a significantly higher expression in CIN tumors. Studies on the involvement of *TRIB3* in the prognosis of GC patients have been reported, albeit with conflicting results [14,15], as well as its role in protecting GC cells from doxorubicin-induced apoptosis [17]. In contrast, only one study reported *TRIB2* to be downregulated in MGC-803 GC cells following treatment with the anti-cancer agent dioscin [16]. Therefore, since the putative role of *TRIB2* in GC is unknown, we focused our attention on *TRIB2* by investigating whether *TRIB2* expression in CIN and MSI tumors correlates with clinicopathological parameters such as sex, tumor stage, and histological grade. Regarding sex, females proved to be prevalent among MSI tumors with low/intermediate *TRIB2* expression; consequently, males mostly fell in the category with the highest *TRIB2* expression. No sex-related distribution in the two *TRIB2* expression groups was observed for CIN GC. No significant association between histologic grade and *TRIB2* expression was found. In contrast, there was a correlation between tumor stage and gene expression levels. Specifically, CIN tumors at advanced stage of progression (T4) mostly showed a lower/intermediate *TRIB2* expression level. On the contrary, MSI tumors at early stages (T2) mostly displayed a high level of *TRIB2* expression. These observations indicate that *TRIB2* downregulation in CIN GCs associates with tumor progression, supporting the notion that this gene has a role as a tumor suppressor in the CIN molecular subtype. We verified this hypothesis through in vitro functional studies by assessing the effect of TRIB2 overexpression in two GC cell lines (MKN45 and NCI-N87) characterized by a CIN phenotype and an “intermediate” endogenous expression of the protein. By different experimental approaches, in both cell lines, we could associate TRIB2 overexpression with suppressed proliferation and colony formation, cell cycle arrest in G2, and reduced migration, thus confirming a tumor-suppressive role of the gene. Some slightly different responses elicited by TRIB2 overexpression in MKN45 and NCI-N87 cells were most likely due to their different genetic backgrounds, including somatic mutations as well as histopathological features of tumors of origin (MKN45 have been derived from a diffuse GC, while NCI-N87 from a GC of the intestinal type).

Several studies demonstrated that TRIB2 regulates different downstream signaling pathways through two regions located at the C-terminus, one required to modulate the MAPK pathway via increased AKT/ERK phosphorylation, and the other that interacts with Ubiquitin E3-ligases and regulates the polyubiquitination of the substrates bound to the pseudokinase domain [8,9,10]. To search for downstream targets mediating the tumor-suppressive effects of TRIB2 in CIN GC cells, we checked for the activation of AKT and ERK upon TRIB2 overexpression. However, we did not observe activation of these two signaling molecules, indicating that *TRIB2* does not exert its tumor suppressor function in CIN GC cells through this specific pathway. In future studies, other TRIB2-mediated carcinogenic mechanisms could be explored, including the degradation of transcription factors (e.g., C/EBPα) via the interaction of TRIB2 with the ubiquitin proteasome system (see below).

*TRIB2* has emerged as an important contributor to malignant hematopoiesis: it induces acute myelogenous leukemia (AML) in mice and is highly expressed in a subset of human AML, exerting its oncogenic role via degradation of C/EBPα transcription factor, a known regulator of cell proliferation [21,22]. *TRIB2* has been established as an oncogene also in some solid tumors. For example, *TRIB2* overexpression has been found in human lung cancers and in non-small lung cancer cells that express low levels of C/EBPα [23]. In melanoma cells, it contributes to malignancy, acting as a repressor of FOXO transcription factors, and its expression correlates with disease stages [23]. In contrast, *TRIB2* has been reported to be a regulator of thymocyte proliferation with tumor suppressor functions, important for the thymopoietic response to genotoxic and oncogenic stress [24]. Tumor-suppressive functions are also supported by findings on ovarian cancer [25] and acute lymphoblastic leukemia (T-ALL) [26]. Regarding the latter, by using a murine model of Notch-induced T-ALL, Stein and colleagues could demonstrate that the deletion of Trib2 decreased the latency and increased the penetrance of T-ALL development. Moreover, in primary murine T-ALL cells, the absence of Trib2 increased C/EBPα expression without altering AKT or ERK phosphorylation [26].

Recent studies support a role for TRIB2 as an emerging novel biomarker of chemoresistance in leukemia, melanoma, osteosarcoma, glioblastoma, and lung and ovarian cancers [12,27]. In cancer cell lines, as well as in vivo models and human samples, *TRIB2* overexpression has been found to mediate resistance against different chemotherapeutic agents and targeted drugs, including 5-fluorouracil, via disruption of the AKT/FOXO and AKT/MDM2/p53 pathways [12].

In GC patients, chemotherapy is performed both pre-operatively as neoadjuvant treatment and after radical surgery as adjuvant treatment to avoid relapse and metastases. GC is commonly treated by using 5-fluorouracil (5-FU) derivatives and platinum combination [28]. However, responsiveness varies among patients, and multidrug resistance after treatment is frequently occurring. Since predictive biomarkers remain lacking, we investigated the effect of TRIB2 overexpression on the responsiveness of GC cells to 5-FU. Several studies have shown that p53 is involved in the response to chemotherapy in GC cells; typically, cell lines with mutated p53 (as NCI-N87) are less sensitive toward 5-FU compared to cells that express wt p53 (as MKN45) [29,30]. At any rate, in both MKN-45 and NCI-N87 cells, the overexpression of *TRIB2* did not appear to alter viability upon treatment, thereby suggesting that *TRIB2* level does not affect the cell response to this chemotherapeutic agent. Altogether, our results point to a tumor-suppressive function of *TRIB2* in GC with a CIN molecular phenotype, identifying *TRIB2* as a possible novel player in CIN GC tumorigenesis. In future studies, it would be interesting to determine the expression levels of TRIB2 in primary human GC samples of the two most relevant molecular subtypes (CIN and MSI) and to correlate them with clinical parameters and therapy response. This will help explore the putative role of TRIB2 as biomarker to stratify patients for disease progression and treatment outcome, and to guide appropriate treatment strategies.

## Figures and Tables

**Table 1:** Percentage of GC samples belonging to the indicated molecular subtypes and organized based on *TRIB* genes expression. The quartile subdivision of gene expression in “high-25” and “other” was used. Cut-off ≥ 0.6 and <0.6 for *TRIB1* and *TRIB2*; cut-off ≥ 0.7 and <0.7 for *TRIB3*. CIN = chromosomal instability, MSI = microsatellite instability, GS = genomically stable, EBV = Epstein–Barr virus tumors, POLE = *POLE* mutated tumors, ultra-mutated phenotype.

	Quartile	CIN (n = 223)	MSI (n = 73)	GS (n = 50)	EBV (n = 30)	POLE (n = 7)
*TRIB1*	High-25	49%	32%	24%	37%	57%
	Others	51%	58%	76%	63%	43%
*TRIB2*	High-25	5%	36%	4%	23%	0%
	Others	95%	64%	96%	77%	100%
*TRIB3*	High-25	43%	23%	4%	10%	43%
	Others	52%	77%	96%	90%	57%

## Data Availability

Data are contained within the article and supplementary materials.

## Supplementary Materials

The following supporting information can be downloaded at: https://www.mdpi.com/article/10.3390/genes15010026/s1, Figure S1: Left panels: *TRIB1* (a), *TRIB2* (b), and *TRIB3* (c) mRNA distribution profiles in the STAD-TCGA. The black arrows indicate the cut-off expressed as z-score for the “high-25”, “low-25”, and “2nd and 3rd” quartiles. Right panels: the box plots show a significant difference in expression levels between high and low quartiles (“high-25” vs. “low-25”, *p* < 0.0001). The *Y*-axis shows mRNA levels expressed in z-scores (log RNA Seq V2 RSEM). Data are shown as mean  ±  standard deviation (SD); **** *p* ≤ 0.0001; Figure S2: *TRIB1* (a), *TRIB2* (b), and *TRIB3* (c) mRNA distribution profiles in the STAD-TCGA: high-25 and other subsets. The black arrows indicate the cut-off expressed as z-score for the “high-25” and “others” subsets (“high-25” vs. “others”, *p* < 0.0001); Figure S3: Correlation between the *TRIB2* mRNA expression levels in CIN and MSI GCs with sex and tumor histologic grade. (a) Sex distribution in tumors with CIN or MSI phenotype in the TCGA-STAD database and high (“high-25”) or intermediate/low (“others”) expression of *TRIB2*. (b) Distribution of CIN or MSI tumors with different histologic grade and high (“high-25”) or intermediate/low (“others”) expression of TRIB2 (TCGA STAD database). G1 to G3: less aggressive and well differentiated to more aggressive and poorly differentiated. GX: grade not determined; Figure S4: Mitogen-activated protein kinase (MAPK) pathway analysis. Western blot of MAPK proteins: total-AKT, phospho-(p)-AKT (Ser473), total-ERK, and phospho-(p)-ERK expression levels in MKN45 and NCI-N87 parental, GFP OE, and TRIB2 OE cell lines. α-tubulin is the internal control. P: parental; GFP OE: GFP overexpressing control cells; TRIB2 OE: TRIB2 overexpressing cells. (b) Quantification of the bands corresponding to the Western blots shown in panel a normalized to a-tubulin; Figure S5: Effect of TRIB2 OE on the response of GC cells to 5-FU. MKN45 (a) and NCI-87 (b) cells overexpressing GFP (GFP OE; control) or overexpressing the fusion protein TRIB2-GFP (TRIB2 OE) were treated with different doses of 5-FU for 72 h. Cell viability was determined by WST-1. Data are shown as mean  ±  standard deviation (SD).
