# Supplementary material for: Tribbles Genes in Gastric Cancer: A Tumor-Suppressive Role for TRIB2
Source: Genes (Basel). 2023 Dec 23;15(1):26. doi: 10.3390/genes15010026 (PMC10815672; doi:10.3390/genes15010026)
Supplement: Supplementary file 1 [file genes-15-00026-s001.zip › genes-2773399-supplementary.pdf]

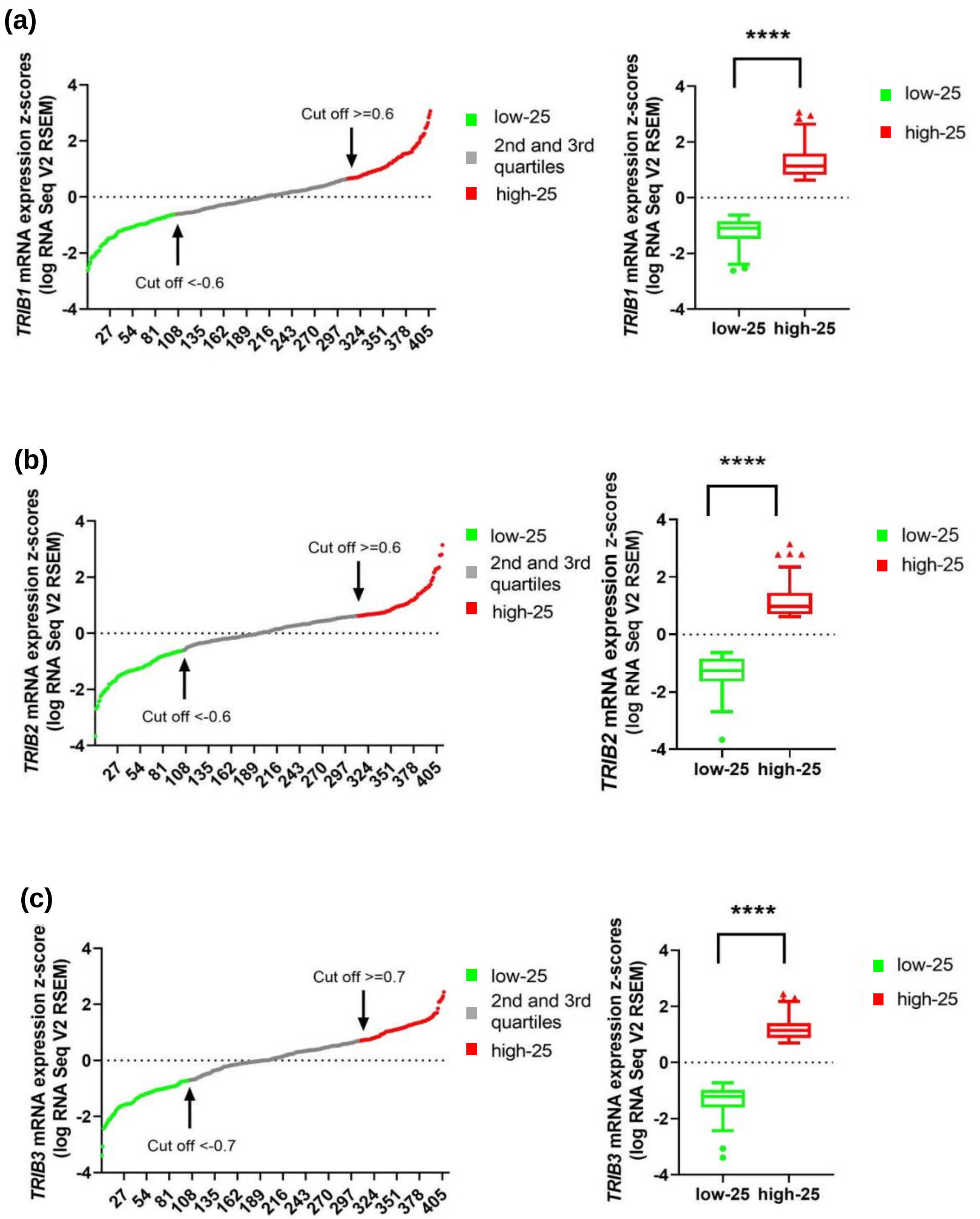

Figure S1. Left panels: *TRIB1* **(a)**, *TRIB2* **(b)** and *TRIB3* **(c)** mRNA distribution profiles in the STAD-TCGA. The black arrows indicate the cut-off expressed as z-score for the “high-25”, “low-25” and “2nd and 3nd” quartiles. Right panels: the box plots show a significant difference in expression levels between high and low quartiles (“high-25” *vs.* “low-25”, $P < 0.0001$). The y-axis shows mRNA levels expressed in z-scores (log RNA Seq V2 RSEM). Data are shown as mean ± standard deviation (SD); **** $P \leq 0.0001$.

(a)

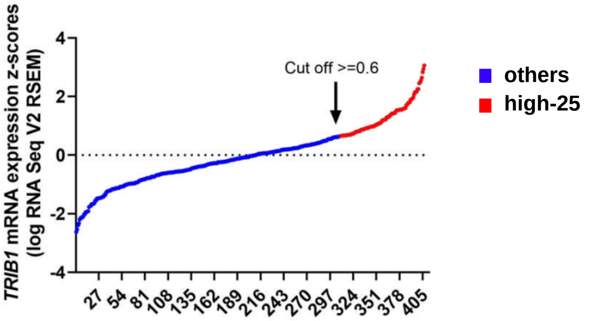

(b)

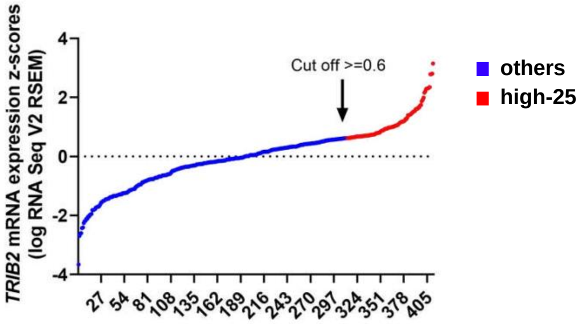

(c)

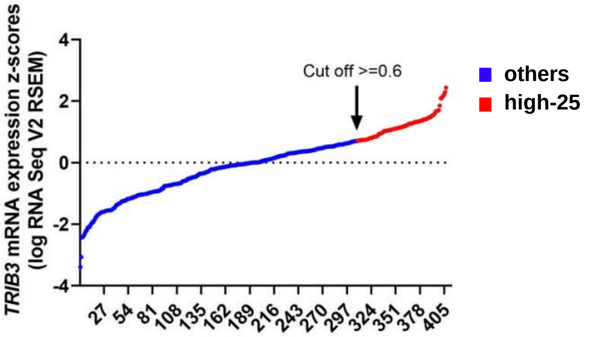

Figure S2. *TRIB1* **(a)**, *TRIB2* **(b)**, and *TRIB3* **(c)** mRNA distribution profiles in the STAD-TCGA: high-25 and others subsets. The black arrows indicate the cut-off expressed as z-score for the “high-25” and “others” subsets (“high-25” *vs.* “others”, $P < 0.0001$).

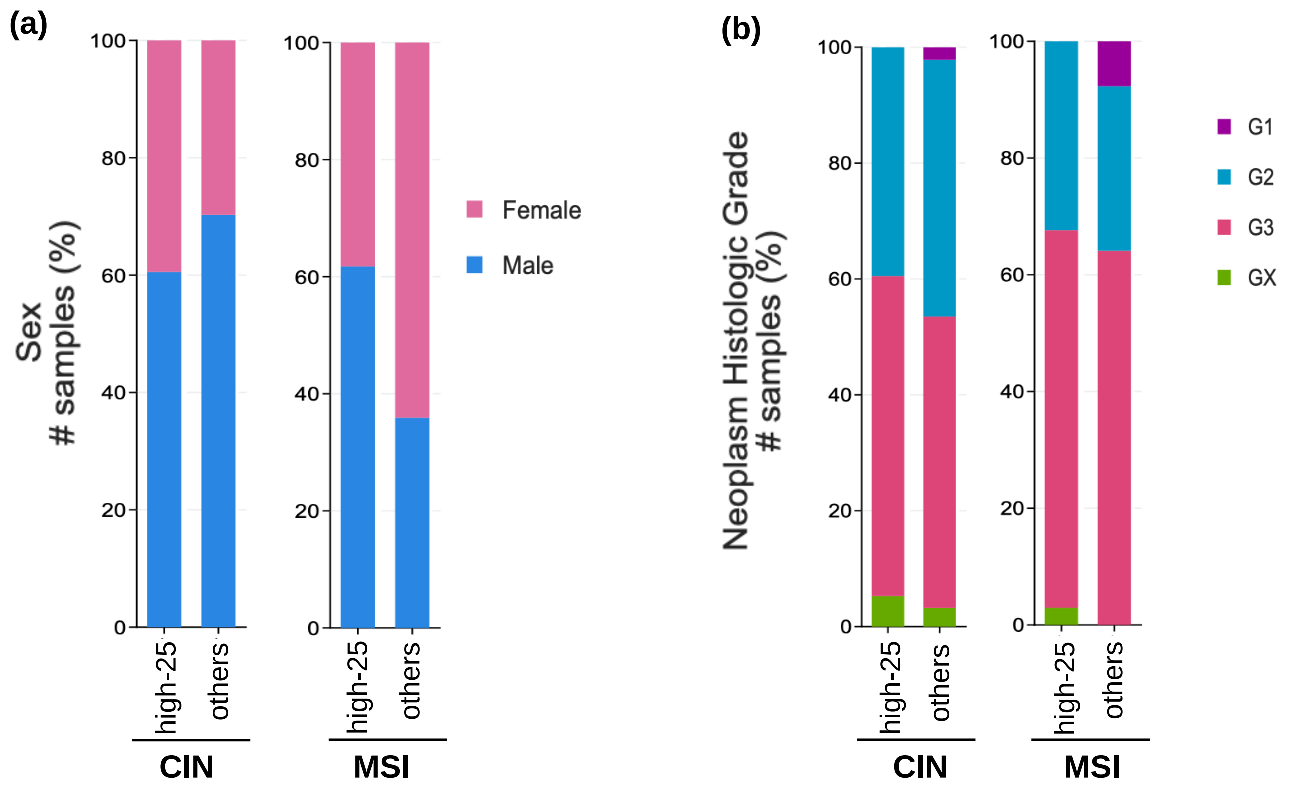

Figure S3. Correlation of *TRIB2* mRNA expression levels in CIN and MSI GCs with sex and tumor histologic grade. (**a**) Sex distribution in tumors with CIN or MSI phenotype in the TCGA-STAD database and high ("high-25") or intermediate/low ("others") expression of *TRIB2*. (**b**) Distribution of CIN or MSI tumors with different histologic grade and high ("high-25") or intermediate/low ("others") expression of *TRIB2* (TCGA STAD database). G1 to G3: less aggressive and well differentiated to more aggressive and poorly differentiated. GX: grade not determined.

(a)

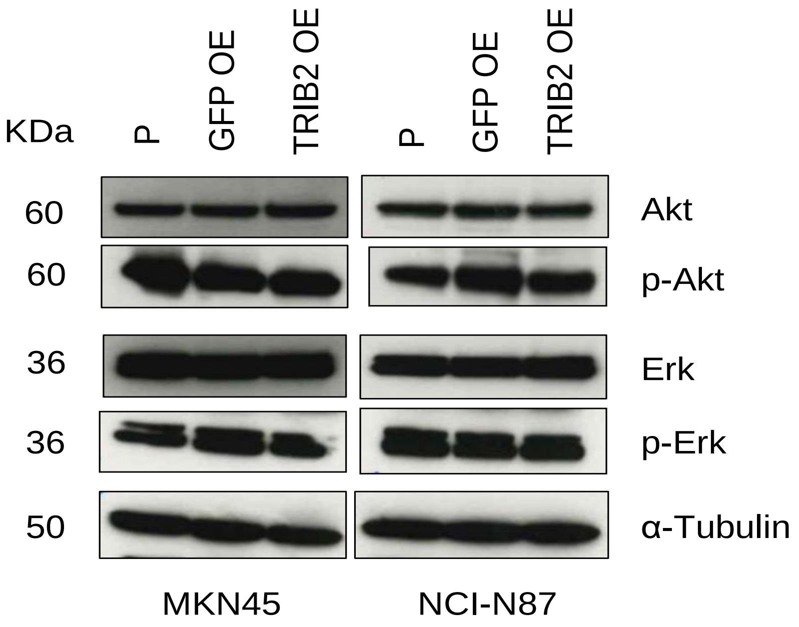

(b)

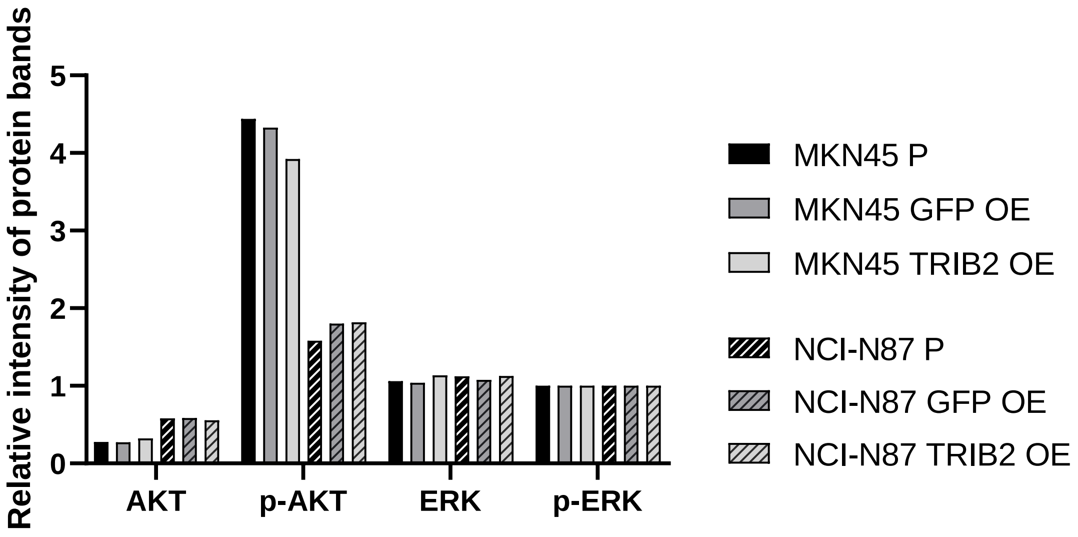

Figure S4. Mitogen-activated protein kinase (MAPK) pathway analysis. **(a)** Western blot of MAPK proteins: total-AKT, phospho-(p)-AKT (Ser473), total-ERK and phospho-(p)-ERK expression levels in MKN45 and NCI-N87 parental, GFP OE and TRIB2 OE cell lines. α-tubulin is the internal control. P: parental; GFP OE: GFP overexpressing control cells; TRIB2 OE: TRIB2 overexpressing cells. **(b)** Quantification of the bands corresponding to the western blots shown in panel a normalized to α-tubulin.

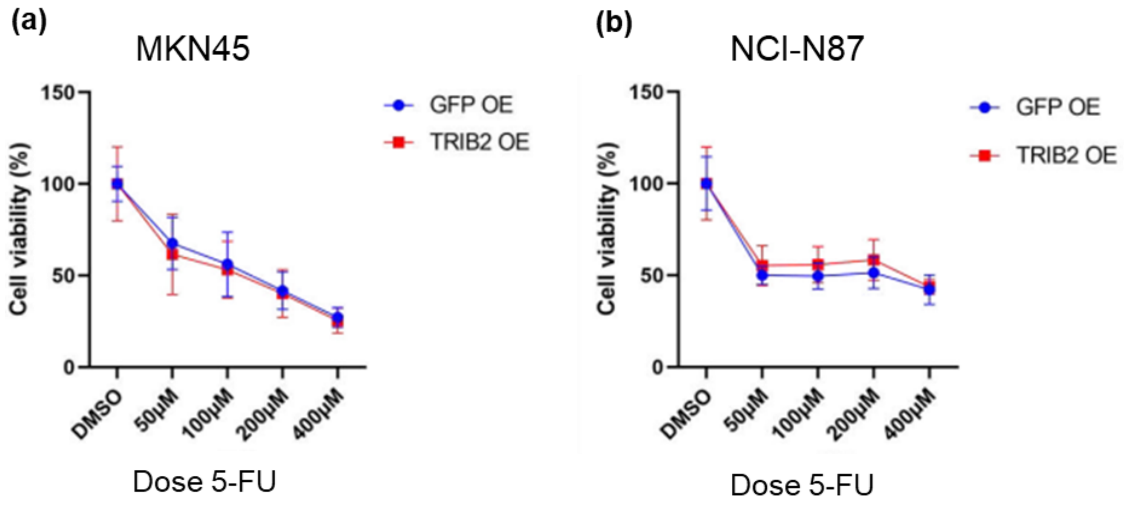

Figure S5. Effect of TRIB2 OE on the response of CIN GC cells to 5-FU. MKN45 **(a)** and NCI-87 **(b)** cells overexpressing GFP (GFP OE; control) or overexpressing the fusion protein TRIB2-GFP (TRIB2 OE) were treated with different doses of 5-FU for 72h. Cell viability was determined by WST-1. Data are shown as mean ± standard deviation (SD).
